# Bilateral Lateral Ventricle Cerebrospinal Fluid Diversion in a Rare Case of Foramen of Monro Occlusion: A Case Report

**DOI:** 10.7759/cureus.95051

**Published:** 2025-10-21

**Authors:** Francisco Rivera, Arnau Benet

**Affiliations:** 1 Neurosurgery, California Neurosurgical Specialists, Thousand Oaks, USA

**Keywords:** cerebrospinal fluid, endoscopic third ventriculostomy, foramen of monro, hydrocephalus, normal pressure hydrocephalus, shunt, ventriculoperitoneal

## Abstract

Obstructive hydrocephalus in young adults is a rare and often misdiagnosed condition. Normal pressure hydrocephalus (NPH), typically affecting older adults, may resemble obstructive forms when bilateral foramen of Monro occlusion is present. Traditional cerebrospinal fluid (CSF) diversion may be insufficient in cases with isolated ventricles. We present a unique case of a 37-year-old patient with presumed NPH, a prior endoscopic third ventriculostomy (ETV), and persistent symptoms due to undiagnosed foraminal obstruction. The patient presented with progressive gait instability and memory impairment. Imaging demonstrated ventriculomegaly with lateral ventricular isolation. An initial right ventriculoperitoneal shunt (VPS) provided partial relief, but persistent symptoms prompted a second procedure. A left VPS was placed and connected to the original system using a single programmable valve via a novel suboccipital tunneling technique, referred to as the “neckband technique.” This case illustrates the diagnostic challenges of differentiating NPH from obstructive hydrocephalus in young adults. It also demonstrates a safe, effective technique for bilateral ventricular drainage using a single valve system. This strategy reduces long-term hardware burden while ensuring effective CSF diversion in patients with bilateral ventricular isolation.

## Introduction

Hydrocephalus is an abnormal accumulation of cerebrospinal fluid (CSF) in the ventricular system affecting approximately 85 out of every 100,000 individuals worldwide. The prevalence of hydrocephalus amongst the elderly exceeds 400 per 100,000 [1]. The causes of hydrocephalus include obstruction, impaired absorption, or, more rarely, hypersecretion of CSF [2]. Hydrocephalus is generally categorized into four principal types: obstructive, communicating, hypersecretory, and normal-pressure hydrocephalus (NPH) [3]. Despite significant advances, the presentation and causes of hydrocephalus in young adults remain incompletely understood, highlighting a critical gap in the current literature.

Normal pressure hydrocephalus (NPH) classically presents in the elderly and, less frequently, in infants [4]. Clinical presentation broadly consists of insidious gait dysfunction, typically shuffling, broad-based, emerging first and most prominently after age 40, often preceding subcortical cognitive decline and urinary urgency or incontinence [5]. The cornerstone of NPH treatment remains CSF diversion with an adjustable valve, while repeated lumbar punctures or acetazolamide are reserved for non-surgical candidates [5]. In patients with obstructive hydrocephalus, management strategies include ventriculoperitoneal shunt (VPS) and endoscopic third ventriculostomy (ETV) [6]. In cases such as colloid cysts or pineal region tumors causing ventricular obstruction, endoscopic techniques have gained prominence, offering the ability to both address the primary lesion and restore CSF dynamics through procedures like ETV [6]. ETV is an established treatment for obstructive hydrocephalus; however, restenosis over time is well known and can result in recurrence [7,8]. In such cases, CSF diversion may be a suitable option to address symptomatic hydrocephalus [9].

We present a case highlighting the complex management of CSF diversion in a 37-year-old patient misdiagnosed with NPH and a prior history of ETV. Additionally, a novel technique was employed to place a bilateral shunt system utilizing a unilateral single valve.

## Case presentation

**Figure 1:**
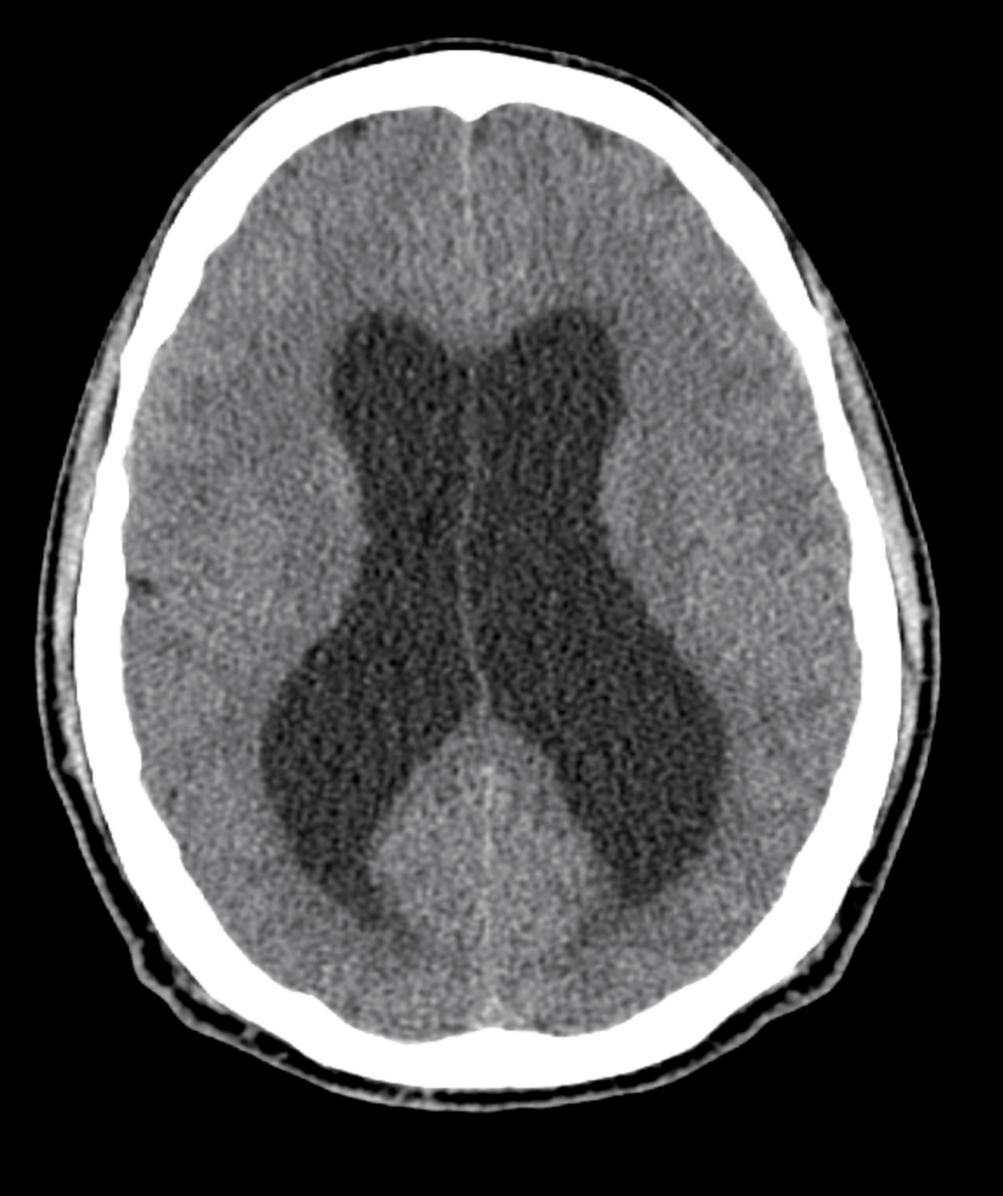
Axial head CT demonstrating hydrocephalus with enlargement of the lateral ventricles.

A 37-year-old male with no relevant family or genetic history presented with progressive memory deterioration and gait instability described as “wobbliness.” His medical history was notable for herpes zoster infection 10 years prior. Early symptoms resembling trochlear nerve palsy had previously delayed the diagnosis of NPH. Seven years prior, he underwent successful ETV for NPH. He remained stable until progressive symptoms recurred several months before admission. Computed tomography (CT) showed ventriculomegaly with crowding of the cisternal space (Figure 1). Trial administration of acetazolamide led to partial symptomatic improvement, reinforcing the diagnosis of recurrent hydrocephalus. No secondary causes such as infection, hemorrhage, or neoplasm were identified despite thorough evaluation. On examination, the patient demonstrated cognitive slowing and broad-based gait instability, without focal motor or sensory deficits. Written consent to publish was obtained, and Institutional Review Board or ethics committee approval was not sought for this case report, as it describes a single clinical case without experimental intervention, and all identifying patient information has been anonymized.

**Figure 2:**
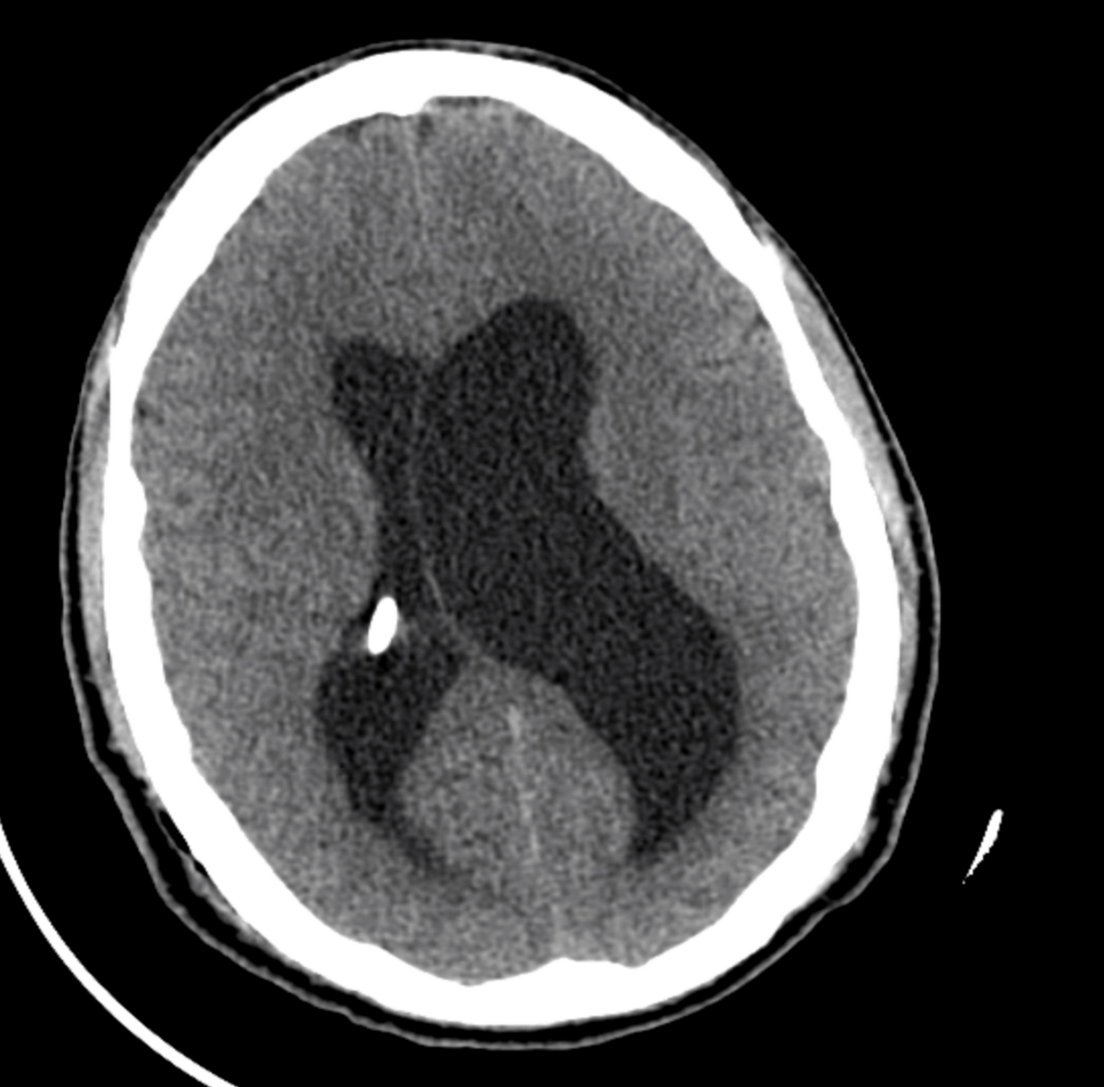
Postoperative axial CT following right ventriculoperitoneal shunt placement, showing selective decompression of the right ventricle and rightward midline shift, consistent with left ventricular isolation.

The patient underwent right-sided VP shunt placement through a parieto-occipital trajectory using neuronavigation for proximal catheter insertion. The catheter immediately released CSF under high pressure (opening pressure >20 mmHg), confirming the diagnosis of hypertensive hydrocephalus. A Certas valve without a syphon was programmed at level 4. Immediate postoperative CT revealed ventricular isolation (Figure 2). Given the persistent symptoms and imaging findings, a second surgical intervention was scheduled. A left parieto-occipital ventricular catheter was placed on a second stage. The distal catheter was attached to the intraventricular catheter with an in-line connector and tunneled suboccipitally, running below the superior nuchal line to minimize mechanical obstruction and patient discomfort while in the supine position. Both right and left proximal catheters were connected to a single programmable valve using a Y-connector system.

**Figure 3:**
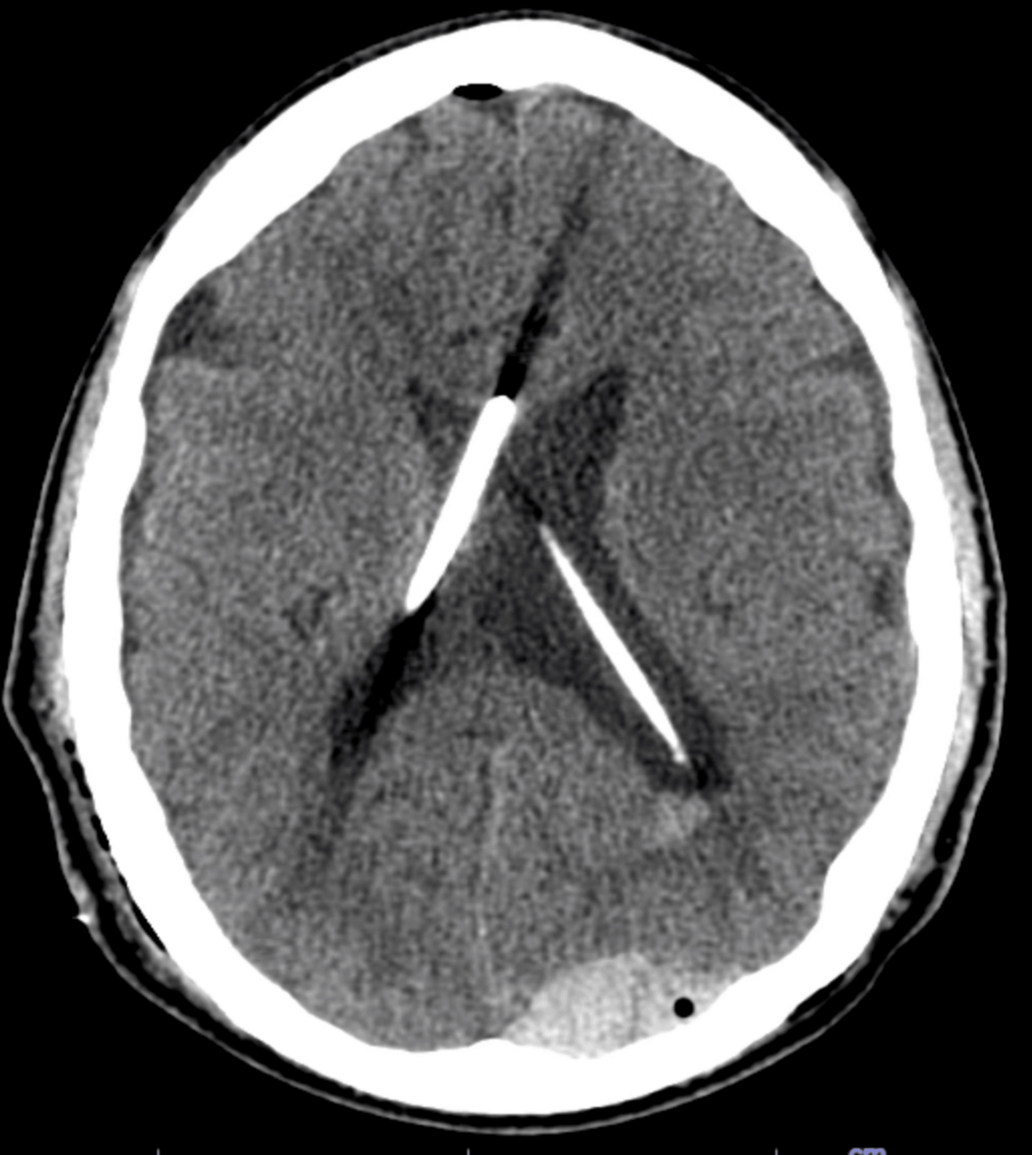
Axial CT obtained after left ventricular catheter placement, confirming bilateral catheter position and restoration of ventricular symmetry.

**Figure 4:**
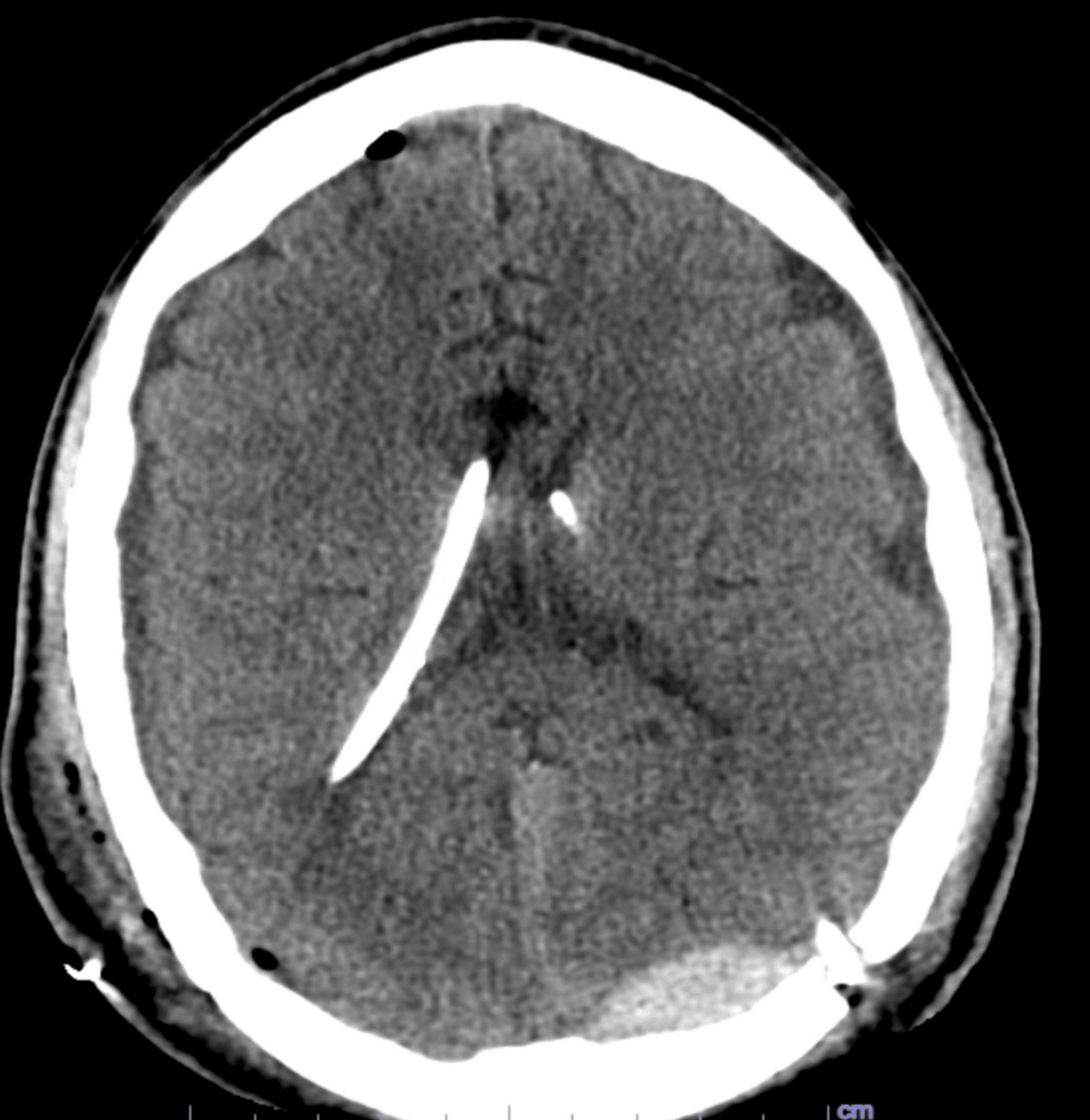
Postoperative axial CT scan showing bilateral ventricular catheter placement with evidence of overdrainage, demonstrated by collapsed lateral ventricles.

Immediate postoperative imaging confirmed accurate placement of the left catheter (Figure 3). On postoperative day one, after left-sided shunt placement, the patient developed headaches when standing up, and CT imaging revealed ventricular overdrainage (Figure 4). Therefore, the valve was adjusted to position 6, resulting in the resolution of symptoms. The patient was discharged on postoperative day five. At follow-up, he reported marked improvement in both cognitive and gait function with no new neurological deficits.

## Discussion

This case underscores the diagnostic challenges of distinguishing true NPH from obstructive hydrocephalus secondary to bilateral foramen of Monro stenosis in young adults. The patient’s age and lack of a clear secondary cause made NPH less likely, and symptom resolution after previous ETV suggested an obstructive process. Furthermore, post-shunt imaging showed lateral ventricular isolation, inconsistent with a communicating hydrocephalus. Intraoperatively, both shunt placements revealed persistently elevated intraventricular pressures, further contradicting NPH. All these factors collectively pointed toward an alternative diagnosis of obstructive hydrocephalus resulting from foraminal obstruction rather than primary NPH. Furthermore, we illustrate a novel surgical strategy consisting of bilateral ventricular catheter placement, converging into a single programmable valve through a “neckband” tunneling technique.

Bilateral foramen of Monro stenosis or occlusion is a rare neurosurgical condition that can lead to hydrocephalus and lateral ventricle isolation. The etiology can be varied, including idiopathic membranous occlusion, true stenosis, or unilateral stenosis with septum deviation causing functional bilateral occlusion [10]. Clinical presentation typically involves headache, which may be dull and burning in nature, with memory impairment and gait disturbances [11,12]. Treatment approaches include endoscopic interventions such as septum pellucidotomy, foraminoplasty, or membrane perforation, but in cases where these fail or are not feasible due to risk of fornix injury, ventriculoperitoneal shunting becomes necessary [10]. Our patient might have been considered for such endoscopic interventions; however, given his history of prior ETV, the persistence of symptoms after the initial shunt placement, and a shared discussion with the patient and family, the team ultimately decided to proceed with placement of a contralateral shunt. In cases of bilateral occlusion, a single shunt may be insufficient due to lateral ventricular isolation, necessitating bilateral shunting procedures, as we evidenced in our patient, where, after the first shunt was placed, the hydrocephalus in the left ventricle was still present [13]. Although colloid cysts represent the most classic lesion causing foraminal obstruction, other intraventricular masses must also be considered. In the absence of an overt lesion, potential acquired causes include post-infectious ependymal scarring, prior hemorrhage, or congenital narrowing. Although the patient had a previous ETV, scarring along the tract was considered unlikely given the seven-year interval and lack of symptomatic recurrence. With no history of relevant illness or injury, the obstruction was ultimately deemed idiopathic.

Traditional VP shunt placement in one ventricle alone would have inadequately managed the CSF burden in this patient. Thus, we present a novel approach to bilateral shunt placement using a single valve instead of two separately. The development of ipsilateral drainage during the initial procedure prompted a thorough reassessment for potential non-communication between the lateral ventricles [14]. By employing a single valve, although carrying a higher procedural complexity, we aimed to enable unified drainage regulation, avoiding the complexity of managing two independent systems for a lifetime (more than 60 years of life expectancy). Therefore, we describe a proof of concept of the "neckband technique," which involves tunneling a second shunt catheter beneath the inion and along the superior nuchal line for rare cases needing bilateral VPS. The novelty of this technique does not lie in the Y-connector itself, but in the suboccipital “neck-band” tunneling below the superior nuchal line, which minimizes mechanical obstruction and patient discomfort in the supine position. Careful suboccipital dissection is critical to preserve muscular, vascular, and neural structures [15]. The catheter is positioned superficial to the majority of the suboccipital muscles but deep to the sternocleidomastoid.

We acknowledge certain limitations in this report. Additional imaging, such as MRI, would have provided a more detailed assessment of the ventricular anatomy and possible obstruction sites. Moreover, a postoperative skull X-ray was performed to verify the correct valve position and catheter course, but these images are not included here. Likewise, while a more detailed description of the suboccipital tunneling technique would enhance the technical understanding, our intent was to emphasize the clinical decision-making and management aspects of the case. A dedicated technical report with comprehensive intraoperative images and illustrations is currently being prepared to complement this publication.

## Conclusions

This case illustrates the importance of re-examining diagnostic assumptions in patients who fall outside the usual demographic profile of normal-pressure hydrocephalus. In young adults, the recurrence of ventriculomegaly after ETV should prompt consideration of secondary obstruction, particularly at the foramina of Monro. Distinguishing true communicating hydrocephalus from subtle obstructive variants is essential, as treatment paradigms and long-term outcomes differ significantly.

Beyond its diagnostic value, this report introduces a practical surgical concept: bilateral ventricular drainage through a single programmable valve using a suboccipital “neckband” route. By simplifying the system while maintaining physiologic control of CSF dynamics, this approach may reduce mechanical burden and lifetime management complexity in select cases of ventricular isolation. More broadly, it underscores that innovation in neurosurgery often arises from the thoughtful adaptation of established principles to unique anatomical and clinical challenges.
